# Renal Contributions to Age‐Related Changes in Mineral Metabolism

**DOI:** 10.1002/jbm4.10517

**Published:** 2021-06-03

**Authors:** Debra L Irsik, Wendy B Bollag, Carlos M Isales

**Affiliations:** ^1^ Charlie Norwood VA Medical Center Augusta GA USA; ^2^ Department of Neuroscience and Regenerative Medicine Augusta University Augusta GA USA; ^3^ Department of Physiology Augusta University Augusta GA USA; ^4^ Division of Endocrinology, Department of Medicine Augusta University Augusta GA USA

**Keywords:** CALCIUM, FIBROBLAST GROWTH FACTOR 23, PARATHYROID HORMONE, PHOSPHATE, VITAMIN D

## Abstract

Aging results in a general decline in function in most systems. This is particularly true with respect to the skeleton and renal systems, impacting mineral homeostasis. Calcium and phosphate regulation requires tight coordination among the intestine, bone, parathyroid gland, and kidney. The role of the intestine is to absorb calcium and phosphate from the diet. The bone stores or releases calcium and phosphate depending on the body's needs. In response to low plasma ionized calcium concentration, the parathyroid gland produces parathyroid hormone, which modulates bone turnover. The kidney reabsorbs or excretes the minerals and serves as the final regulator of plasma concentration. Many hormones are involved in this process in addition to parathyroid hormone, including fibroblast growth factor 23 produced by the bone and calcitriol synthesized by the kidney. Sclerostin, calcitonin, osteoprotegerin, and receptor activator of nuclear factor‐κB ligand also contribute to tissue‐specific regulation. Changes in the function of organs due to aging or disease can perturb this balance. During aging, the intestine cannot absorb calcium efficiently due to decreased expression of key proteins. In the bone, the balance between bone formation and bone resorption tends toward the latter in older individuals. The kidney may not filter blood as efficiently in the later decades of life, and the expression of certain proteins necessary for mineral homeostasis declines with age. These changes often lead to dysregulation of organismal mineral homeostasis. This review will focus on how mineral homeostasis is impacted by aging with a particular emphasis on the kidney's role in this process. © 2021 The Authors. *JBMR Plus* published by Wiley Periodicals LLC on behalf of American Society for Bone and Mineral Research.

## Introduction

Aging is marked by a general decline in function that spans multiple organ systems. This is a highly complex and often variable process that can be further exacerbated by the confounding effects of disease. One of the most obvious age‐related changes in women is menopausal loss of endogenous estrogen and the associated skeletal protection afforded by the hormone.^(^ ^1^ ^)^ Aging also sees an increase in oxidative stress and inflammation.^(^ ^2^, ^3^ ^)^ There is increased cellular senescence,^(^ ^4^, ^5^ ^)^ and the regenerative ability of the organism decreases with age.^(^ ^6^ ^)^ In humans, there are decreases in the growth hormone/insulin‐like growth factor‐1 axis with aging, although perhaps somewhat paradoxically, reduced axis activity is associated with a longer life span.^(^ ^7^, ^8^, ^9^ ^)^ On the other hand, increased insulin resistance has a negative impact on life span, and caloric restriction promotes longevity,^10^, ^11^ ^)^ in part by improving insulin sensitivity.

An age‐related decline in kidney function begins in the fourth decade of life,^(^ ^12^, ^13^ ^)^ characterized by hemodynamic changes such as decreased glomerular filtration rate (GFR) and renal blood flow, and leads to uremia, an accumulation of toxins in the blood. As renal function worsens, inflammatory cytokines increase and are associated with progression to dialysis.^(^ ^14^ ^)^ These functional changes may be a by‐product of structural changes such as decreased renal cortical mass, tubulointerstitial fibrosis, decreased glomerular number and size, and/or increased glomerulosclerosis.^(^ ^15^, ^16^ ^)^ Decreased afferent arteriolar resistance increases glomerular capillary hydrostatic pressure, potentially precipitating proteinuria,^(^ ^17^ ^)^ a hallmark of chronic kidney disease.

Bone^(^ ^18^ ^)^ and muscle mass^(^ ^19^ ^)^ decrease with aging because of several factors, including the inefficiency of continuous bone remodeling and lack of physical activity. Mechanical loading–induced bone formation is decreased with aging.^(^ ^20^ ^)^ Women experience increased susceptibility for bone loss in the postmenopausal period. Aging decreases the number of osteoblasts and their function.^(^ ^21^ ^)^ Bone marrow stromal cells (BMSC) also decrease with age,^(^ ^22^ ^)^ and they become senescent, losing the ability to become osteoblasts. As bone mass decreases with aging, bone marrow fat increases^(^ ^23^ ^)^ and secretes inhibitors of bone formation (such as tumor necrosis factor‐α and interleukin 6). In addition, systemic inflammation with aging, inflammaging,^(^ ^2^, ^24^, ^25^ ^)^ results in increased mobilization of calcium from the bone.^(^ ^26^ ^)^ This chronic negative calcium balance, when severe, diminishes bone strength and may lead to osteoporosis.^(^ ^26^ ^)^ Osteoporosis predisposes to microcrack formation in the bone, which then further contributes to the inflammatory response.

Changes in bone formation and resorption can also result in alterations in calcium and phosphate homeostasis. Plasma concentrations of calcium and phosphate are tightly regulated by a complex signaling network involving bone, parathyroid glands, intestine, and kidney. Because 99% of calcium^(^ ^27^, ^28^ ^)^ and 85% of phosphate^(^ ^29^ ^)^ in the body are stored in the bone, it can be viewed as a reservoir for these minerals. Bone is, in fact, an active player in the homeostasis of calcium and phosphate concentration, both receiving signals from parathyroid hormone (PTH) as well as sending information to the kidney through fibroblast growth factor‐23 (FGF23). PTH is produced by the parathyroid glands in response to decreased extracellular concentration of circulating ionized calcium, whereas the intestine absorbs both minerals from the diet. The kidney adjusts reabsorption and excretion of these minerals to maintain appropriate plasma levels in concert with these other mechanisms that promote absorption in the gastrointestinal tract or redistribution from storage. The kidney also produces calcitriol, the active form of vitamin D (1,25‐dihydroxyvitamin D_3_), another important factor in mineral homeostasis and critical for intestinal absorption of calcium and phosphate.

Because the kidney serves as an ultimate regulator of plasma calcium and phosphate concentration, a decline in kidney function due to aging or disease can greatly impair mineral homeostasis. This review will focus on how the kidney and bone interact to maintain mineral homeostasis and will examine the impact of aging on this process.

## Calcium Homeostasis

### Role of the intestine in absorption of dietary calcium

Calcium is an important mineral not just for bone health but also for the role it plays in cell signaling and other processes. Therefore, cellular calcium concentration is tightly regulated. In addition, calcium in the plasma can modulate cell excitability, and calcium imbalances can disrupt nerve transmission, cardiac rhythm, and muscle contraction.

The recommended daily allowance of calcium is 1000 mg for adults but rises to 1200 mg in women after age 50 years.^(^ ^30^ ^)^ Many people become calcium deficient because of an age‐related increased incidence of lactose intolerance resulting in decreased consumption of calcium‐rich (and vitamin D‐fortified) dairy products,^(^ ^27^, ^31^ ^)^ poor diet, or interactions with other medications.^(^ ^32^ ^)^ The recommended daily allowance of calcium rises to 1200 mg for all individuals over 70 years to offset these challenges.^(^ ^30^ ^)^ However, many people fall short of achieving the recommended intake. One review of controlled clinical trials suggests that there is no benefit of increasing dietary calcium intake or taking calcium supplements in terms of fracture risk in healthy individuals.^(^ ^33^ ^)^ Therefore, the full impact of low calcium intake remains to be determined.

Gastrointestinal calcium absorption is influenced by the calcium concentration in the intestinal lumen, flow rate, and solubility. An acidic environment increases the absorption of calcium ions. The lumen of the duodenum, with a pH of 6.0, allows maximal calcium solubility.^(^ ^34^ ^)^ Only 40% of our dietary intake is absorbed into the blood with the majority excreted in feces (800 mg/d, a combination of absorbed and secreted).^(^ ^28^ ^)^

Dietary calcium is absorbed in the intestine through paracellular and transcellular pathways. Passive paracellular transport occurs in the small intestine (duodenum, jejunum, and ileum), in which calcium moves from the high concentration in the lumen through tight junctions into the interstitial space to be taken up into the blood. Tight junctions between intestinal enterocytes are composed of occludins and claudins. Claudins 2, 12, and 15 are involved in intestinal calcium transport through charge selectivity.^(^ ^35^ ^)^ Claudins are regulated by calcitriol and transcellular proteins,^(^ ^35^ ^)^ suggesting a compensatory upregulation of paracellular transport in the absence of effective transcellular transport. The sodium/hydrogen exchanger (NHE3) provides the driving force for paracellular transport. NHE3^−/−^ mice exhibit dysregulation of renal and intestinal calcium handling and loss of cortical bone density. Despite increased calcitriol levels, NHE3^−/−^ mice display decreased expression of claudins 2 and 15 but not 12.^(^ ^36^ ^)^

Calcium can also be absorbed through the energy‐dependent transcellular pathway in which calcium moves against its concentration gradient (intracellular to interstitial). Calcium entry through the enterocyte's apical membrane involves one of three channels: the transient receptor potential vanilloid subfamily members 5 or 6 (TRPV5, TRPV6) channels or the voltage‐gated L‐type calcium channel Ca_v_1.3. TRPV5 and TRPV6 are highly selective for calcium and are constitutively active at various concentrations of calcium and at physiologic membrane potentials.^(^ ^37^ ^)^ TRPV6 is highly expressed in the duodenum and is thought to be the major route for transcellular calcium entry. Active calcium absorption decreases by 60% in TRPV6 knockout mice.^(^ ^38^ ^)^ TRPV6 is activated by hyperpolarization and may be responsible for calcium transport under polarized conditions such as in between meals or during starvation.^(^ ^39^, ^40^ ^)^ The L‐type calcium channel Ca_v_1.3, which is highly expressed in the jejunum and ileum, may be most important under depolarized conditions during digestion.^(^ ^39^, ^40^ ^)^ The third channel, TRPV5, has a lower expression in the intestine but may compensate when TRPV6 is dysfunctional.

Calbindins are calcium‐binding proteins that transport calcium through the cytosol of the intestinal cells to the basolateral membrane to enhance calcium absorption. The main form found in mammalian intestines is calbindin‐D_9K._^(^ ^41^ ^)^ Calbindins also buffer intracellular calcium concentration to prevent cellular toxicity. Calcium can be pumped out of the cell through the basolateral membrane via the plasma membrane Ca‐ATPase (PMCA1b) with ATP expenditure or it can be exchanged for three sodium ions through the sodium‐calcium exchanger (NCX1).^(^ ^41^ ^)^

Calcitriol, produced by the kidney from circulating 25‐hydroxyvitamin D_3_, aids in the absorption of calcium. It binds the vitamin D receptor (VDR) in the cytosol, and in a complex with the retinoic acid receptor (RXR),^(^ ^42^ ^)^ translocates to the nucleus, where it acts as a transcription factor binding to vitamin D response elements in target genes such as TRPV6,^(^ ^41^ ^)^ claudins,^(43^ ^)^ and calbindins.^(^ ^44^ ^)^ The induced expression and activity of these proteins then promote calcium uptake. Lithocholic acid, a bile acid, is also a VDR ligand and has been shown to increase paracellular calcium absorption.^(^ ^45^ ^)^ PTH does not have any known direct action in the intestine but influences calcium absorption by stimulating renal proximal tubule synthesis of calcitriol.

The ability to absorb calcium in the intestine decreases with age^(^ ^46^, ^47^, ^48^ ^)^ in part due to the kidneys' decreased synthesis of calcitriol and increased production of the enzyme that degrades it, CYP24A1.^(^ ^49^, ^50^ ^)^ This, in turn, would decrease expression of calcitriol‐dependent proteins such as TRPV6 and calbindin D_9K,_^(^ ^51^ ^)^ possibly accounting for lower calcium absorption in the postmenopausal state.^(^ ^52^ ^)^

### Calcium concentration in the blood

Blood contains 1% of the calcium in the body, and the plasma calcium concentration is tightly regulated. In the plasma, calcium can be bound to proteins (41%) or to anions (9%) or be freely ionizable (50%).^(^ ^53^ ^)^ Under acidic conditions, less calcium is bound to proteins, whereas with higher pH, calcium binding increases.^(^ ^28^ ^)^ A low concentration of circulating ionized calcium in the plasma stimulates the release of PTH, which in turn stimulates bone resorption and release of calcium from bone, indirectly increases intestinal absorption through stimulating the formation of calcitriol in the renal proximal tubule, and increases renal reabsorption in the thick ascending limb of the loop of Henle (TAL) and distal nephron. Decreased intestinal calcium absorption with aging could perturb blood calcium concentration if not compensated. However, the kidney reabsorbs calcium until it is unable to maintain the appropriate concentration and then compensation occurs at the expense of the bone, leading to bone loss.

### Role of bone in storage and release of calcium

Bone has the main functions of providing structural support, enabling movement and storing minerals. After the skeleton is fully formed and has reached maturity, the bone undergoes continuous regeneration through the process of remodeling. This remodeling serves to repair damage to the bone caused by wear and tear. Bone remodeling requires a balance between formation (osteoblasts) and resorption (osteoclasts). However, this is an inefficient process that results in a continuous decline in bone mass from the time of epiphysial plate closure.^(^ ^21^, ^54^ ^)^

Most of the calcium (99%) in our bodies resides in bone. Calcium ions form hydroxyapatite crystals with phosphate ions, although it is thought there is some free calcium or amorphous calcium‐phosphate crystals at the surface of bone as well. Osteoblasts secrete collagen and ground substance (and a number of hormones) to form the matrix component of bone that is ultimately mineralized. Bone mineralization occurs after osteoblast secretion of alkaline phosphatase, the activity of which promotes mineral deposition on the polymerized collagen strands. Bone mineralization is regulated through the opposing forces of pyrophosphate (PP_i_), nucleotide pyrophosphatase/phosphodiesterase type 1 (NPP1), ankylosis protein (ANK), and tissue non‐specific alkaline phosphatase (TNAP). TNAP promotes mineralization by degrading PP_i_, an inhibitor of hydroxyapatite crystallization, to inorganic phosphate to allow hydroxyapatite crystal formation. In osteoblasts, NPP1 and ANK increase extracellular levels of PP_i_ by hydrolyzing ATP to PP_i_ and allowing PP_i_ transport through the plasma membrane, respectively.^(^ ^55^, ^56^ ^)^ Inhibitors such as PP_i_, as well as magnesium, fetuin A, osteoprotegerin, and vitamin K–dependent matrix gla‐protein, are present in the plasma and extra‐skeletal tissues to prevent deposition of calcium crystals outside of the bone.^(^ ^53^, ^57^ ^)^

**Fig. 1:**
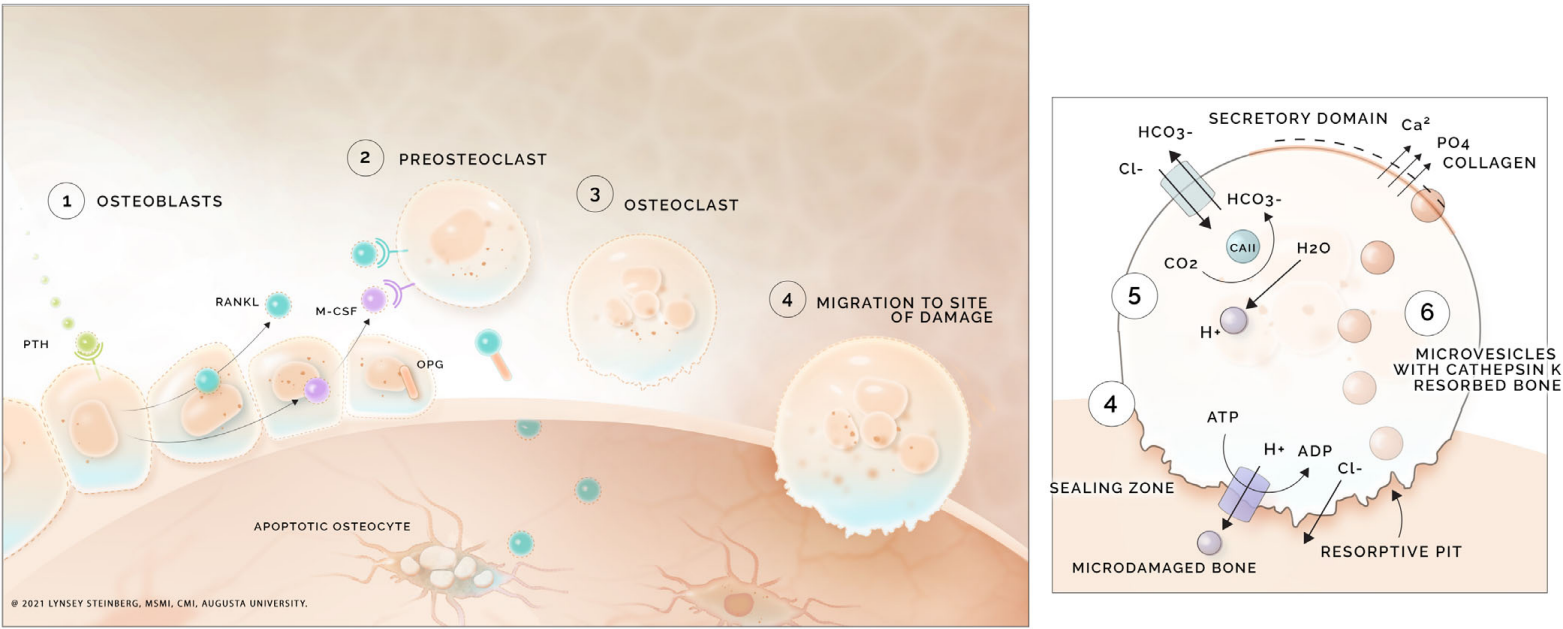
Osteoclastogenesis and bone resorption. (1) Parathyroid hormone (PTH) binds parathyroid hormone receptor (PTHR) on osteoblasts, inducing release of receptor activator of NF‐κB ligand (RANKL) and macrophage colony‐stimulating factor (M‐CSF). Osteoblasts also produce osteoprotegerin (OPG), which acts as a decoy receptor for RANKL. (2) RANKL and M‐CSF bind their respective receptors on preosteoclasts and induce differentiation to osteoclasts. (3) Mature osteoclasts migrate to the site of damage on bone. (4) The cell becomes polarized and forms a sealing zone around the damaged area. The plasma membrane forms a ruffled border. Acidification of the damaged area occurs through hydrogen (H‐ATPase) and chloride (chloride channel) transport. Demineralized bone is packaged into microvesicles with cathepsin K and released from the cell through the functional secretory domain.

Osteoclasts are responsible for releasing calcium into the bloodstream through the process of bone resorption (Fig. 1). {FIG1} Through a poorly understood mechanism, osteocytes recruit osteoclasts to sites of remodeling via receptor activator of nuclear factor‐κB ligand (RANKL) release by osteoblasts.^(^ ^58^ ^)^ Activated, mature osteoclasts adhere to bone and undergo a cytoskeletal rearrangement, resulting in polarization of the cell membrane to form an apical membrane consisting of a ruffled border and sealing zone. The sealing zone separates the site of resorption from the rest of the bone surface.^(^ ^59^ ^)^ The osteoclasts then secrete cathepsin K, a lysosomal protein, and MMP9 to degrade collagen and hydrochloric acid to dissolve matrix and mineral. The degradation products are endocytosed, and calcium, phosphate, and collagen are released into the extracellular fluid through the basolateral membrane.

Major regulators of bone resorption are PTH, calcitriol, calcitonin, and estrogen. PTH can have both anabolic and catabolic effects on bone depending on the dose and periodicity.^(^ ^60^ ^)^ PTH has no direct effects on osteoclasts in humans because they lack the PTH receptor, PTH1R. Instead, high, continuous‐dose PTH stimulates osteoblasts to release RANKL. RANKL binds its receptor, RANK, on preosteoclast cells, which induces them to differentiate into mature osteoclasts. PTH also inhibits the expression of osteoprotegerin, which is secreted by osteoblasts and acts as a decoy receptor to bind RANKL and oppose resorption; therefore, high, sustained levels of PTH enhance RANKL action on resorption. On the other hand, low‐dose, intermittent PTH promotes bone formation in part by inhibiting sclerostin.^(^ ^60^ ^)^ Sclerostin is produced by osteocytes and inhibits bone formation through Wnt/β‐catenin signaling, preventing osteoblast proliferation, differentiation, and survival.^(^ ^60^ ^)^

Inadequate calcitriol results in the bone diseases rickets in children and osteomalacia in adults. Calcitriol has both direct and indirect effects on the bone. The main function of calcitriol appears to be maintenance of the plasma concentration of calcium and phosphate primarily through intestinal absorption, which indirectly impacts the bone.^(^ ^61^ ^)^ VDR^−/−^ mice exhibit rickets, which can be corrected by feeding a diet enriched with calcium and phosphate. However, there are also direct effects of calcitriol that are not corrected when plasma calcium concentration is restored in the VDR^−/−^ mice.^(^ ^62^ ^)^ These include decreased numbers of osteoblasts and osteoclasts, decreased mineral apposition rate, and decreased expression of RANKL.

Calcitonin, which is made in the thyroid by parafollicular cells, opposes PTH action to increase plasma calcium concentration, acting to prevent hypercalcemia. Calcitonin binds its receptor on osteoclasts to inhibit resorption.^(^ ^63^ ^)^ However, under conditions of high, sustained PTH, such as in primary hyperparathyroidism, calcitonin is unable to inhibit PTH‐stimulated resorption. Similarly, estrogens decrease bone resorption by promoting osteoclast apoptosis,^(^ ^64^ ^)^ consistent with the increased resorption observed after menopause and the resulting decreased estrogen levels.

Aging reflects a state of increased resorption and decreased bone formation due in part to a reduced number of osteoblasts and a decreased ability of BMSCs to differentiate to form osteoblasts. These deficiencies lead to reduced bone mass (trabecular and cortical),^(^ ^65^ ^)^ which is further exacerbated by the loss of estrogen in the postmenopausal state. Although men also lose bone and develop osteoporosis with age, elderly women are particularly vulnerable to this and are at greater risk for fracture.

### Role of the kidney in reabsorption and excretion of calcium

**Fig. 2:**
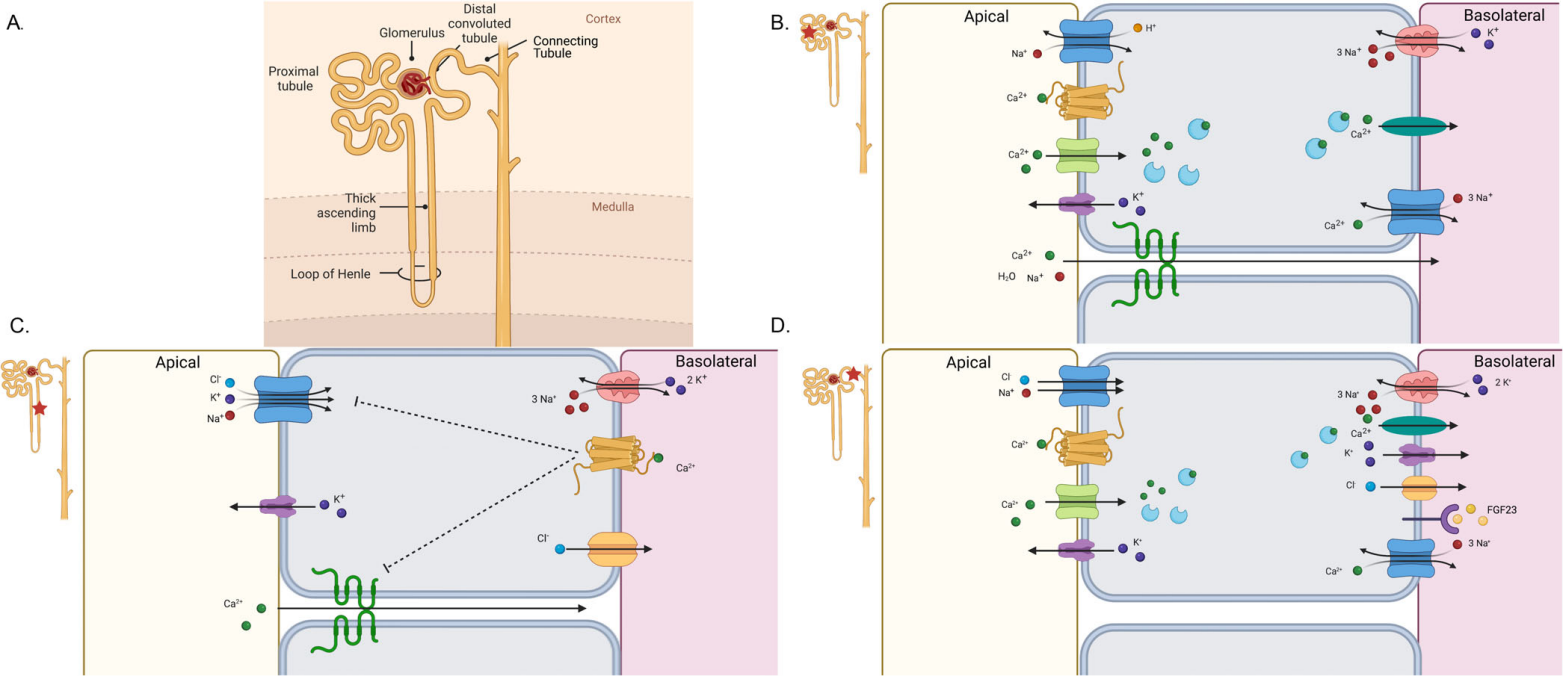
Renal calcium transport. (*A*) Depiction of the nephron and its segments: proximal tubule, thick ascending limb of the loop of Henle (TAL), distal convoluted tubule/connecting tubule (DCT/CNT). (*B*) Calcium transport in the proximal tubule. Apical membrane: NHE3 (blue), CaSR (gold), TRPC3 (green), K+ channel (purple). Basolateral membrane: Na/K ATPase (red), PMCA1 (green), NCX1 (blue). Intracellular transport occurs through calbindin D_28K_ (light blue). Paracellular transport occurs through claudins 2, 10a, and 17 (green). (*C*) Calcium transport in the TAL. Apical membrane: NKCC2 (blue), ROMK (purple). Basolateral membrane: Na/K ATPase (red), CaSR (gold), chloride channel (ClC‐Kb, gold). Paracellular transport occurs through claudins 10, 14, 16, and 19 (green). (*D*) Calcium transport in the DCT/CNT. Apical membrane: NCC (blue), CaSR (gold), TRPV5 (green), ROMK (purple). Basolateral membrane: Na/K ATPase (red), PMCA4 (green), K channel (purple), ClC‐Kb (gold), FGFR/Klotho (purple), NCX1 (blue). There is no paracellular transport in this segment.

The kidneys are a metabolically active tissue that receive 20% of cardiac output. They filter 125 mL/min of blood in the average adult with their main functions to remove waste, maintain extracellular fluid volume, and produce functionally important substances such as the hormones, erythropoietin, and calcitriol. The functional unit of the kidney is the nephron composed of the glomerulus and the tubule (Fig. 2*A*). {FIG2} The glomerulus, the site of blood filtration, is composed primarily of capillaries. The filtrate enters the proximal tubule, where the majority of reabsorption occurs. The tubule is made up of epithelial cells, with the various segments of the tubule containing different cell types and regulating the reabsorption of molecules based on the receptors, channels, and transporters that each cell type expresses. Mineral transport occurs primarily in the proximal tubule, TAL, distal convoluted tubule (DCT), and connecting tubule (CNT). Filtrate moves through the lumen of the tubule, and various substrates enter the epithelial cells through channels and transporters on the apical membrane, as well as via a paracellular pathway. Ions are transported through the cell and exit through the basolateral membrane into the interstitial fluid from which they can reenter the bloodstream. Some substances can also enter the tubule lumen without being filtered through the process of secretion. As the filtrate reaches the end of the tubule, it is concentrated in the collecting duct to create the final urine to be excreted.

Free calcium can be filtered and reabsorbed in the kidney but cannot be secreted,^(^ ^53^ ^)^ whereas calcium bound to protein cannot be filtered. Most of the filtered calcium is reabsorbed in the proximal tubule (60%)^(^ ^66^ ^)^ through the passive paracellular pathway (80%).^(28^ ^)^ This pathway is similar to that described in the intestine in which calcium moves down a transepithelial electrochemical gradient from a higher concentration in the tubule lumen to a lower concentration in the interstitial fluid. This pathway is PTH‐ and vitamin D–independent. Calcium moves through the tight junctions with water and sodium, and claudins 2, 10a, and 17 regulate this process.^(^ ^53^ ^)^ Claudin 2, a cation‐selective isoform, has been shown to be particularly important for calcium homeostasis, as deletion in mice leads to a reduction in calcium reabsorption and increased delivery of calcium to the distal nephron, resulting in hypercalciuria and stone formation.^(^ ^67^ ^)^ NHE3 on the apical membrane and Na/K ATPase on the basolateral membrane provide the driving force for passive transport (Fig. 2*B*).

The remaining 20% of proximal tubule reabsorption occurs through the transcellular pathway, although the mechanism has not yet been elucidated (Fig. 2*B*). It may involve the calcium‐sensing receptor (CaSR), which has been recently detected in the proximal tubule.^(^ ^68^ ^)^ The CaSR is a class C G‐protein–coupled receptor for which calcium ions act as ligand^(^ ^69^ ^)^ and is responsible for monitoring extracellular calcium levels to regulate PTH release from the parathyroid chief cells.^(^ ^70^ ^)^ The crystal structure of this receptor reveals a large extracellular domain capable of binding calcium.^(^ ^71^, ^72^ ^)^ Under conditions of high extracellular calcium concentration, CaSR in the parathyroid gland couples to either G_q/11_ or G_i/o_. G_q/11_ activates phospholipase C, resulting in increased levels of diacylglycerol and inositol trisphosphate (IP_3_), which binds to IP_3_ receptors on the endoplasmic reticulum to release calcium from this storage site, thereby increasing intracellular calcium concentration and inhibiting the release of PTH.^(^ ^69^ ^)^ G_i/o_ inhibits adenylyl cyclase, which decreases cyclic AMP, leading to decreased activation of cAMP‐dependent protein kinase (PKA) and ultimately inhibiting gene transcription. Deletion of CaSR is embryonically lethal, underscoring the importance of this signaling pathway in calcium homeostasis.^(^ ^73^ ^)^

In the proximal tubule as well, activation of CaSR on the apical membrane of proximal tubule cells may activate the phospholipase C pathway causing IP_3_‐mediated release of intracellular calcium stores from the endoplasmic reticulum. Apical transient receptor potential canonical member 3 (TRPC3) may then be stimulated to further increase intracellular calcium through calcium influx.^(^ ^74^ ^)^ Calcium then exits the cell through NCX1 or PMCA1 on the basolateral membrane (Fig. 2*B*). Deletion of TRPC3 in knockout mice increased urine calcium excretion as well as stone formation.^(^ ^74^ ^)^

Approximately 25% to 30% of calcium reabsorption occurs in the TAL (Fig. 2*C*). Paracellular transport in this region depends on claudins 10, 14, 16, and 19^(^ ^53^ ^)^ and the driving force is provided by the sodium gradient generated through the apical sodium, potassium, chloride co‐transporter type 2 (NKCC2), and the basolateral Na/K ATPase.^(^ ^28^ ^)^ The recycling of potassium into the lumen through the renal outer medullary potassium channel (ROMK) creates a positive lumen potential. The highest levels of CaSR protein expression in the kidney were detected in the TAL.^(^ ^68^ ^)^ Paracellular transport in the TAL is regulated in part by the CaSR in the basolateral membrane, possibly through modulation of the claudins^(^ ^75^ ^)^ to inhibit permeability. Inhibition of CaSR in parathyroidectomized rats increased calcium reabsorption in the TAL and increased the plasma concentration of calcium without impacting bone.^(^ ^75^ ^)^ In the TAL, how the CaSR affects paracellular transport is only partially understood; identifying the exact targets of CaSR signaling in this segment will be important.

Fine‐tuning of calcium reabsorption occurs in the distal nephron, which accounts for up to 15% of total reabsorption.^(^ ^76^ ^)^ Calcium reabsorption in the DCT and CNT occurs solely through transcellular transport (Fig. 2*D*). Calcium enters the cell through TRPV5 located in the apical membrane. CaSR co‐localizes with TRPV5 in this region, and TRPV5 activity is increased with CaSR activation.^(^ ^76^ ^)^ The CaSR is also found in the basolateral membrane in the DCT, although its function there is unclear.

Renal‐specific calbindin D_28K_ in the cytosol buffers the intracellular calcium concentration and transports calcium to the basolateral membrane where the ion exits the cell through either PMCA4 or NCX1. TRPV5 expression is increased by PTH and calcitriol. The CaSR has been shown to increase sodium reabsorption through the sodium chloride co‐transporter (NCC) via the With‐no‐lysine kinase 4 (WNK4)‐SPS1‐related proline/alanine‐rich kinase (SPAK) pathway.^(^ ^77^ ^)^

FGF23 has also been shown to stimulate calcium reabsorption through TRPV5 by increasing the abundance of the channel in the apical membrane.^(^ ^78^ ^)^ This enhancement is regulated through FGF23 binding to its receptors FGFR and αKlotho and inducing downstream signaling events involving extracellular signal‐regulated protein kinase 1/2 (ERK 1/2), serum/glucocorticoid‐regulated kinase 1 (SGK1), and WNK4.

With aging, GFR is often decreased, although this is highly variable among individuals. Chronic kidney disease is defined as the point at which 50% of normal function is lost. However, differentiating between the normal loss of function with aging and disease‐induced loss is difficult. Irrespective of the cause, as GFR decreases, the kidneys are less efficient in regulating plasma calcium concentration.

## Phosphate Homeostasis

### Role of the intestine in phosphate homeostasis

Phosphate affects acid–base balance, energy homeostasis, and many cellular processes. Phosphate is important for plasma membrane composition (phospholipids), energy transfer (ATP), cell signaling (phosphorylation), nucleic acid backbone structure, and bone strength. Most of the phosphate in the extracellular fluid is in the inorganic form (P_i_), although organic phosphate makes up the majority in the body. Measurement of P_i_ is the standard way of assessing phosphate status, used because it is easier to measure than organic phosphate. Also, it is thought that alterations in P_i_ reflect changes in total body phosphate.^(^ ^79^ ^)^

The average American adult consumes phosphorous in excess of the recommended 700 mg/d.^(^ ^30^ ^)^ It is readily available in its organic form in dairy products, meat, and vegetables and in its inorganic form as food additives such as in cheese and soda. Phytate, the predominant plant‐based organic form of phosphorous, is indigestible in humans.^(^ ^80^ ^)^ Organic animal‐ and non‐phytate plant‐based phosphorous has lower digestibility (40% to 60%) compared with inorganic forms (90%).^(^ ^80^ ^)^

Phosphate is primarily absorbed in the duodenum in humans by an unsaturable passive, paracellular transport process that increases with dietary phosphate consumption. The driving force for transport is provided by apical NHE3. This transporter regulates salt and water absorption and maintains pH in an electroneutral manner, with sodium absorbed in exchange for proton secretion. When NHE3 is inhibited, intracellular proton concentration rises, decreasing intracellular pH. This increased acidity specifically decreases paracellular transport of phosphate by causing a conformational change in tight junction proteins to increase resistance,^(^ ^81^ ^)^ although the claudins involved have not been identified. NHE3 inhibition has become a new strategy to reduce hyperphosphatemia in patients with chronic kidney disease.^(^ ^82^ ^)^ On the other hand, lithocholic acid has been shown to increase paracellular phosphate absorption through suppression of claudin 3 in a VDR‐dependent manner.^(^ ^45^ ^)^

An active transport pathway is available during phosphate deficiency. Most of the active transport occurs through the apical type II sodium‐dependent phosphate co‐transporter 2b (NPT2b, encoded by SLC34A2), the expression of which is regulated by calcitriol.^(^ ^83^ ^)^ Type III sodium‐dependent phosphate transporter 1 (PiT1, encoded by SLC20A1) and 2 (SLC20A2) are ubiquitously expressed but account for less phosphate absorption.^(^ ^83^ ^)^ The basolateral transport mechanism has not been identified yet. Deletion of NPT2b in knockout mice resulted in minimal changes in urinary or fecal phosphate excretion under normal dietary conditions.^(^ ^84^ ^)^ However, with phosphate restriction, NPT2b^−/−^ mice maintained plasma phosphate levels through bone resorption.^(^ ^84^ ^)^

Finally, it has been suggested that matrix extracellular phosphoglycoprotein (MEPE) produced in the bone can directly inhibit phosphate absorption in the intestine under conditions of high dietary phosphate intake.^(^ ^85^, ^86^ ^)^ However, more studies are needed to determine this mechanism.

### Phosphate concentration in plasma

Phosphate concentration is tightly regulated in the blood (1%, 2.5 to 4.5 mg/dL).^(^ ^87^ ^)^ At physiological pH, phosphate exists in the plasma in two unbound forms (85%) with the remaining bound to proteins (15%).^(^ ^29^, ^79^ ^)^ The unbound forms are monohydrogen phosphate (HPO_4_^2−^), which predominates approximately fourfold at physiologic pH, and dihydrogen phosphate (H_2_PO_4_^−^).

The body's ability to detect changes in the extracellular phosphate concentration and respond appropriately is referred to as phosphate sensing. This process is not completely understood but may involve PiT1and PiT2 in the bone and CaSR in the parathyroid gland as extracellular sensors.^(^ ^29^ ^)^ The CaSR has multiple binding sites for phosphate,^(^ ^71^ ^)^ and phosphate binding induces PTH release.^(^ ^88^ ^)^ The major hormones involved in regulating phosphate levels are FGF23, PTH, and calcitriol.

Hyperphosphatemia develops due to renal dysfunction. Plasma phosphate concentration is positively correlated with all‐cause mortality both in patients with chronic kidney disease and those with elevated levels that still fall within normal parameters^(^ ^89^ ^)^ and negatively correlates with life span.^(^ ^90^, ^91^ ^)^ Current therapies for regulating hyperphosphatemia, such as that which occurs with chronic kidney disease, include dietary restriction and phosphate binders.

### Role of the bone in phosphate homeostasis

Phosphate is stored in bone (85%) as hydroxyapatite or amorphous calcium phosphate and in soft tissue (14%), particularly muscle, as creatinine phosphate and ATP.^(^ ^92^ ^)^ Phosphate exits the extracellular fluid and enters the bone for the purpose of bone mineralization. Phosphate from the extracellular fluid is transferred to osteoblasts through PiT1 and 2, where it is packaged into microvesicles with calcium and orphan phosphatase (PHOSPHO1) and released by exocytosis to the matrix.^(^ ^79^ ^)^ TNAP and NPP1 regulate both P_i_ availability as well as the concentration of PP_i_.^(^ ^79^ ^)^ FGF23 inhibits bone mineralization by binding to its receptor FGFR3 on osteoblasts and inhibiting TNAP transcription.^(^ ^93^ ^)^ Phosphate and calcium homeostasis are coupled in the bone because breakdown of hydroxyapatite releases both.

FGF23, a major regulator of phosphate concentration, is produced by osteocytes and osteoblasts as a result of chronic increases in extracellular phosphate concentration. The sensing mechanism for this effect has not been fully elucidated, although it may involve PiT2 and its ability to mediate FGF23 synthesis and secretion.^(^ ^94^ ^)^ In transgenic knockout mice, PiT2 gene ablation prevented release of FGF23 despite changes in phosphate load and independent of PTH or calcitriol.^(^ ^94^ ^)^ On the other hand, low calcitriol concentration is known to increase FGF23 production by osteoblasts independently of PTH,^(^ ^95^ ^)^ although PTH can increase FGF23 release,^(96^ ^)^ whereas, in contrast, FGF23 decreases PTH secretion.

FGF23 is synthesized as a 251‐amino acid protein that undergoes cleavage of the 24‐amino acid signal peptide to form secreted intact FGF23.^(^ ^97^ ^)^ Secreted FGF23 can be regulated both through post‐translational modification and cleavage. Thus, intact FGF23 is stabilized through O‐glycosylation on Thr178 by GALNT3;^(^ ^96^ ^)^ it can be cleaved by the serine endoprotease furin at S180 to form the cleaved peptide, which is thought to be inactive and/or inhibitory.^(^ ^96^ ^)^ Humans that lack the ability to cleave FGF23 develop hypophosphatemic rickets (autosomal dominant or X‐linked).^(^ ^95^ ^)^ One of the receptors for FGF23, FGFR1c, may also be involved with phosphate sensing through intracellular phosphorylation, independent of ligand.^(^ ^98^ ^)^ In the kidney, FGF23 acts to promote phosphate excretion.

### Role of the kidney in phosphate homeostasis

The kidney is responsible for maintaining phosphate homeostasis through excretion and reabsorption. Inappropriately high phosphate concentration in the extracellular fluid (hyperphosphatemia) is mainly due to failure of the kidney to excrete phosphate. Phosphate reabsorption in the kidney differs from that of calcium. Phosphate is freely filtered (95%) by the glomerulus. Phosphate is reabsorbed until the transport maximum (0.1 mmol/min) is reached.^(^ ^79^, ^99^ ^)^ After that, the excess is excreted.^(^ ^100^ ^)^ PTH decreases the transport maximum in the kidney to increase Pi excretion.^(^ ^79^ ^)^

**Fig. 3:**
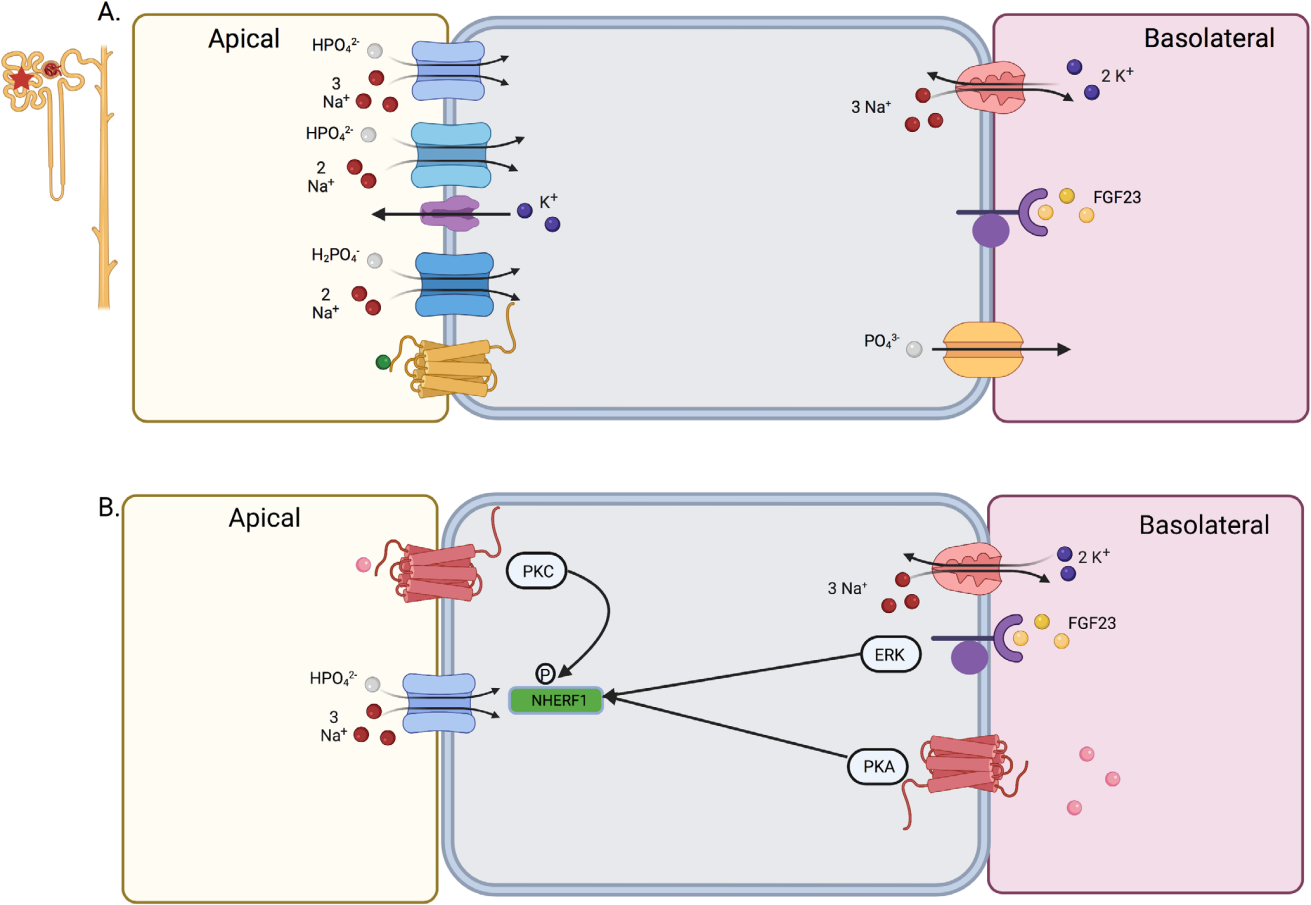
Renal phosphate transport. (*A*) Phosphate transport in the proximal tubule. Apical membrane: NPT2a (light blue), NPT2c (medium blue), K channel (purple), PiT2 (dark blue), CaSR (gold). Basolateral membrane: Na/K ATPase (red), possible phosphate transporter, XPR (gold), FGFR/Klotho (purple). (*B*) Role of NHERF1 in proximal tubule phosphate transport. Apical membrane proteins: PTHR (dark red), NPT2a (light blue). Basolateral membrane: Na/K ATPase (red), FGFR/Klotho (purple), PTHR (dark red). Intracellular protein: NHERF1 (green).

The proximal tubule is responsible for 75% to 80% of reabsorption through the transcellular pathway. This involves the uptake of phosphate through the sodium‐phosphate co‐transporters NPT2a, NPT2c, and PiT2 in the apical membrane and the exit through the basolateral membrane through an unknown mechanism that may involve the xenotropic and polytropic retrovirus receptor 1, XPR1 (Fig. 3*A*).^(^ ^101^ ^)^ {FIG3} NPT2a is electrogenic, transporting three sodium ions for each HPO_4_^2−^ and serves as the main (80%) transporter for reabsorption in the proximal tubules.^(^ ^102^ ^)^ Knockout mice that lack NPT2a exhibit phosphate wasting and hypophosphatemia,^(^ ^103^ ^)^ as well as hypercalcemia with hypercalciuria. Their plasma PTH concentration is decreased, while calcitriol is elevated. On the other hand, NPT2c is electroneutral, transporting two sodium ions for each HPO_4_^2−^ and accounting for 20% of reabsorption. However, deletion of NPT2c in knockout mice did not alter plasma phosphate concentration.^(^ ^104^ ^)^ PiT2 is also electrogenic but transports two sodium ions in exchange for phosphate in the form of H_2_PO_4_^−^.

FGF23 regulates phosphate reabsorption in the proximal tubule. FGF23 binds to its receptor FGFR1c and co‐receptor αKlotho on the basolateral membrane.^(^ ^105^ ^)^ The Klotho gene derived its name from the Greek fate who spun the thread of life because loss of the protein results in premature aging with shortened life span, sarcopenia, bone loss, and vascular calcification.^(^ ^106^, ^107^, ^108^ ^)^ αKlotho is a single‐pass transmembrane protein that helps to stabilize FGF23 binding to FGFRs. αKlotho is mainly found in the basolateral membrane of the proximal tubule and in the distal nephron.^(^ ^109^ ^)^ αKlotho null mice develop hyperphosphatemia due to resistance to FGF23.^(^ ^110^, ^111^ ^)^ In humans, αKlotho decreases with age, resulting in various indicators of vascular aging, including hypertension, vascular calcification, and endothelial dysfunction.^(^ ^108^ ^)^ αKlotho can be cleaved by a disintegrin and metalloproteinase (ADAM) 10 and ADAM 17 to produce a soluble form that is able to circulate;^(^ ^112^ ^)^ however, the importance of the soluble receptor is still being determined. Although initial reports suggest that soluble klotho can induce signaling cascades in extrarenal tissues^(^ ^113^ ^)^ and may play a role in maintaining intact FGF23 levels,^(^ ^114^ ^)^ further research is needed to define its physiologic importance.

FGF23 binding to its receptors causes two main actions. The first is that phosphate reabsorption is decreased due to internalization of NPT2a and NPT2c. This redistribution occurs through a signaling cascade involving ERK1/2, SGK1, and sodium‐hydrogen exchanger regulatory factor 1 (NHERF1).^(^ ^115^ ^)^ NHERF1's role appears to be to traffic NPT2a to and retain it in the apical membrane.^(^ ^116^ ^)^ When NHERF1 is phosphorylated, NPT2a becomes internalized, leading to a reduction in phosphate reabsorption. Indeed, NHERF1^−/−^ mice exhibit hypophosphatemia, phosphate wasting, and internalization of NPT2a.^(^ ^117^ ^)^ Both FGF23 and PTH induce the phosphorylation of NHERF1.^(^ ^118^ ^)^ Apical parathyroid hormone receptor (PTHR) triggers the activation of protein kinase C (PKC), to lead to its phosphorylation, whereas basolateral PTHR elicits PKA activation.^(^ ^119^, ^120^ ^)^ Apical activation of phospholipase C/PKC may be more important in NPT2a internalization.^(^ ^121^ ^)^ Dopamine bound to D1‐like receptors in the proximal tubule activates PKA, which in turn activates PKC to phosphorylate NHERF1.^(^ ^122^ ^)^ The reason why three pathways converge on NHERF1 to induce differential regulation requires further investigation.

The second consequence of FGF23 binding to its receptor is the decreased synthesis of calcitriol due to inhibition of 25‐hydroxyvitamin D 1α‐hydroxylase (encoded by CYP27B1), the enzyme that converts the precursor to the active form of vitamin D, and to upregulation of 25‐hydroxyvitamin D 24‐hydroxylase (encoded by CYP24A1), the enzyme responsible for degradation of the active form. Deletion of FGF23 in knockout mice results in elevated calcitriol levels that can be normalized with deletion of 1 α‐hydroxylase.^(^ ^123^ ^)^

An additional 10% of filtered phosphate may be reabsorbed in the distal nephron^(^ ^124^ ^)^ and the remaining 10% is excreted. However, little is understood about the mechanism of phosphate reabsorption in the distal nephron. Recently, NPT2b has been localized to the apical membrane of the TAL in the kidney, but its purpose requires further investigation.^(^ ^125^ ^)^ Furthermore, data concerning the effect of aging on phosphate homeostasis are limited. However, since phosphate levels increase markedly with chronic kidney failure, and kidney function declines with aging, plasma phosphate levels might be expected to gradually rise with increasing age. Further investigation is needed to determine the aging‐related changes of phosphate handling, as well as the mechanisms involved.

## Conclusion

**Fig. 4:**
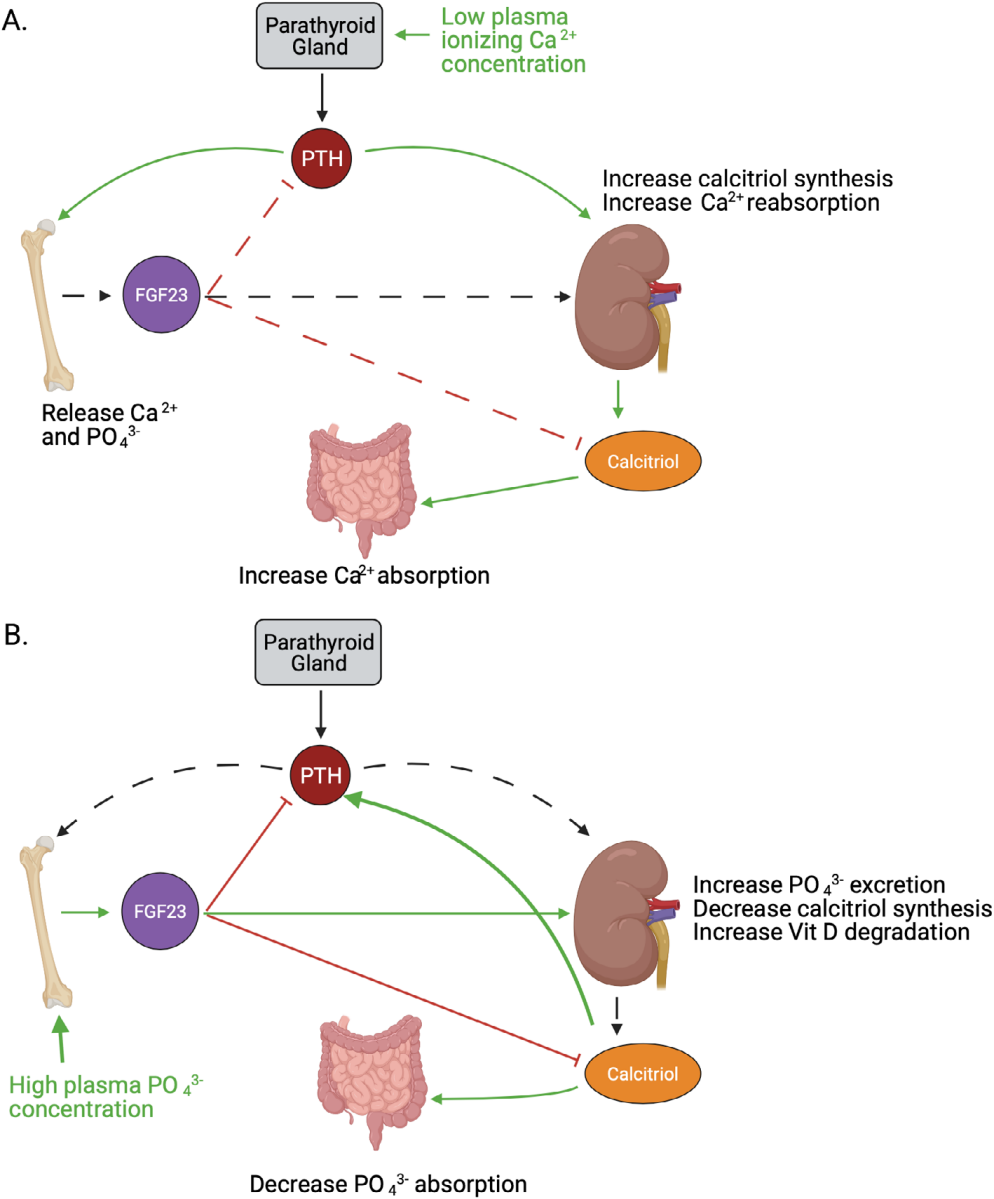
Integrated signaling to maintain mineral homeostasis. (*A*) Low ionized plasma calcium concentration is detected by the parathyroid glands, which secrete parathyroid hormone (PTH). PTH signals to the bone to release stored calcium and phosphate. PTH also promotes calcitriol synthesis in the kidney, which increases calcium absorption in the intestine. FGF23 inhibits PTH and calcitriol synthesis to provide a brake on the system. (*B*) High plasma phosphate concentration is sensed by the bone to release FGF23, which increases phosphate excretion by the kidney and decreases calcitriol synthesis to decrease intestinal phosphate absorption.

Dysregulation of mineral homeostasis can have far‐reaching consequences for the organism as a whole. With changes that occur with aging, the system becomes disrupted. Calcium and phosphate concentrations are interdependent, and the hormones that regulate them (PTH, FGF23, calcitriol) depend on cross‐talk among the intestine, parathyroid glands, bone, and kidneys (Fig. 4). {FIG4} The intestines are less able to absorb calcium in aged individuals, particularly in postmenopausal women. This deficiency will be compensated with reduced calcium excretion in the kidney until plasma concentration can no longer be maintained. At this point, PTH will signal for bone turnover to release both phosphate and calcium stores, weakening bones. Bone formation decreases with aging as well, an effect that is likely multifactorial, although the mechanisms need to be more fully elucidated. The system compensates with increased PTH, which further accelerates bone aging. With aging and cardiovascular disease contributing to chronic kidney disease, which in turn can weaken bones, new strategies to maintain mineral homeostasis are needed. In view of the rapidly rising aged population worldwide, knowledge about the mechanisms regulating these pathways is of great importance as it should help to identify novel therapeutic targets.

## Conflicts of Interest

The authors declare no conflict of interest.

## Author Contributions

**Debra Irsik:** Funding acquisition; writing‐original draft; writing‐review & editing. **Wendy Bollag:** Writing‐review & editing. **Carlos Isales:** Funding acquisition; writing‐review & editing.

### Peer Review

The peer review history for this article is available at https://publons.com/publon/10.1002/jbm4.10517.
